# Supplementary material for: Machine learning-based glucose prediction with use of continuous glucose and physical activity monitoring data: The Maastricht Study
Source: PLoS One. 2021 Jun 24;16(6):e0253125. doi: 10.1371/journal.pone.0253125 (PMC8224858; doi:10.1371/journal.pone.0253125)
Supplement: S1 Fig — (DOCX) [file pone.0253125.s001.docx]

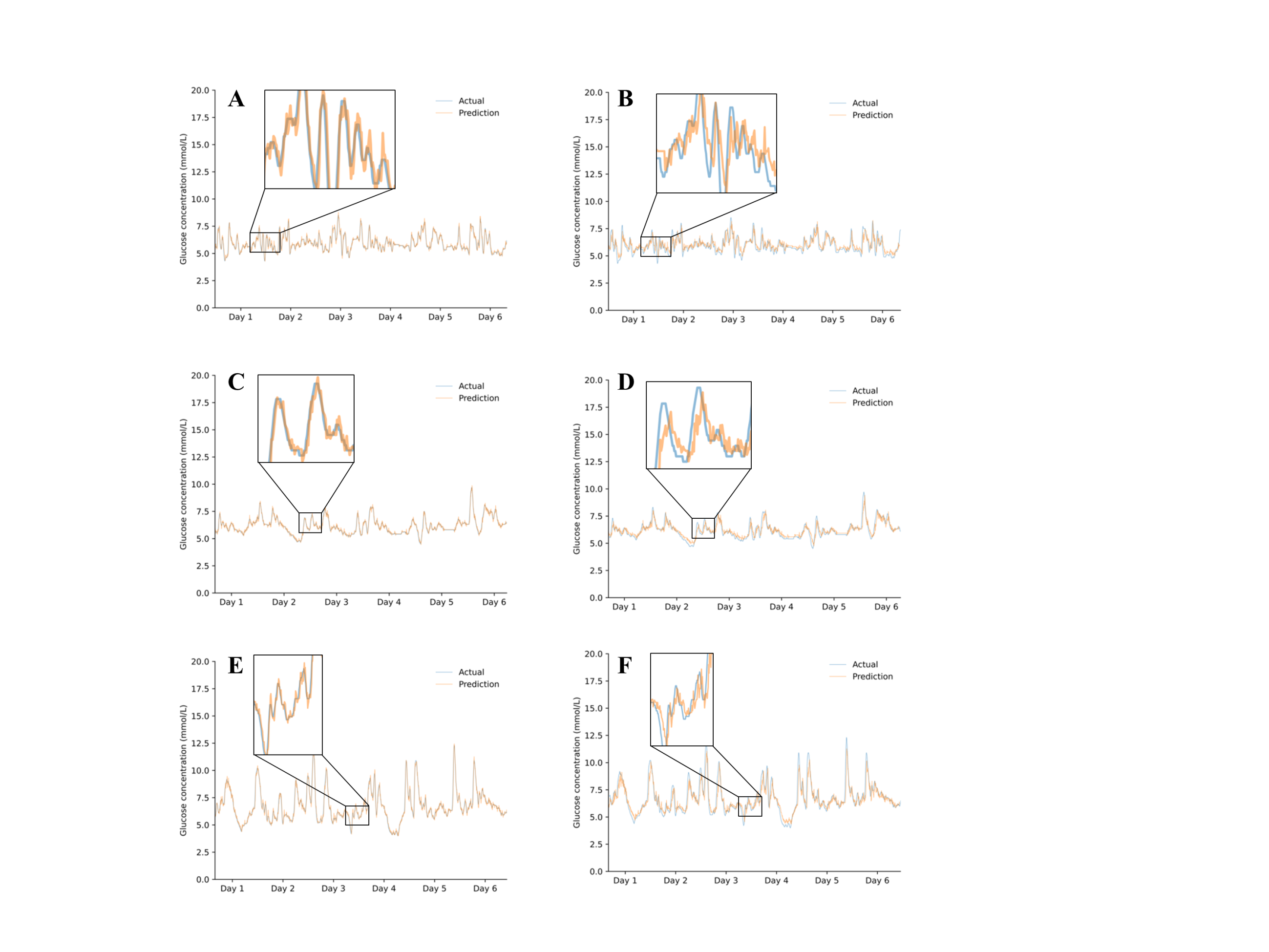


**S1 Fig. Illustrative examples of continuous glucose monitoring-based machine learning model predictions compared to actual values**

Predictions in an individual with normal glucose metabolism (NGM) on a time interval of 15 (A) and 60 (B) minutes. Predictions in an individual with prediabetes (PreD) on a time interval of 15 (C) and 60 (D) minutes. Predictions in an individual with type 2 diabetes (T2D) on a time interval of 15 (E) and 60 (F) minutes.
