# Supplementary material for: Machine learning-based glucose prediction with use of continuous glucose and physical activity monitoring data: The Maastricht Study
Source: PLoS One. 2021 Jun 24;16(6):e0253125. doi: 10.1371/journal.pone.0253125 (PMC8224858; doi:10.1371/journal.pone.0253125)
Supplement: S3 Fig — (DOCX) [file pone.0253125.s003.docx]

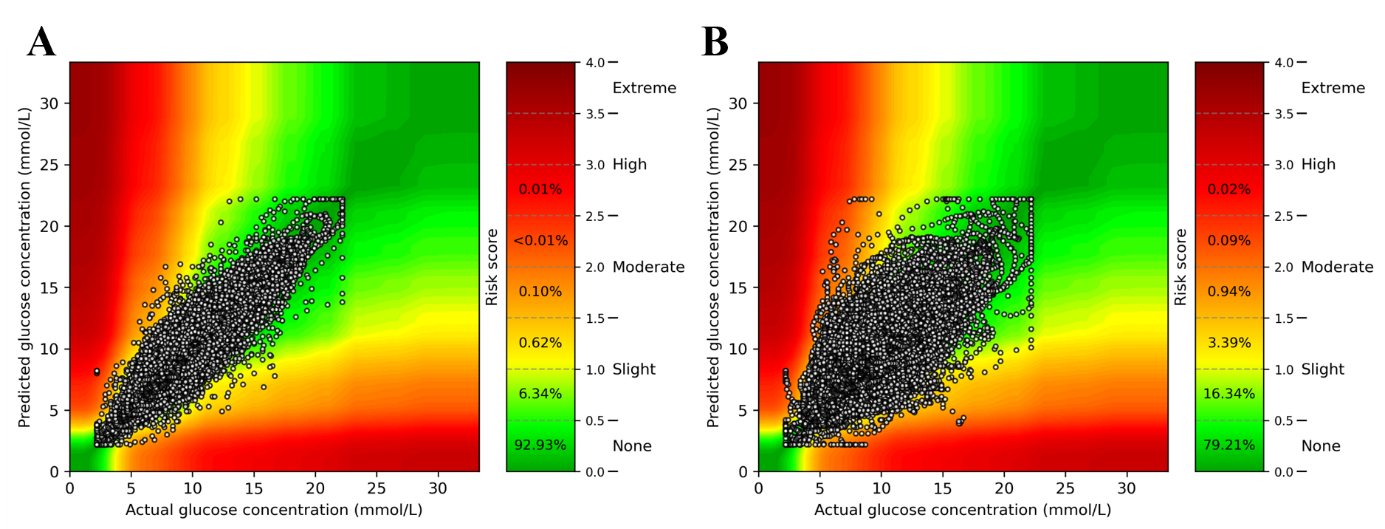


**S3 Fig. Surveillance error grid evaluation of glucose prediction safety at time intervals of 15 and 60 minutes using glucose value t_0_ as predictor**

Assessment of glucose prediction safety in individuals with type 2 diabetes (n=43) at 15 minutes (panel A) and 60 minutes (panel B) using a naïve approach with t0 as predictor. The risk score values translate to the following degrees of risk: 0 - 0.5, none; 0.5 - 1.0, slight (lower); 1.0 - 1.5, slight (higher); 1.5 - 2.0, moderate (lower); 2.0 - 2.5, moderate (higher); 2.5 – 3.0, great (lower); 3.0 - 3.5, great (higher); > 3.5 extreme.
