# Supplementary material for: Machine learning-based glucose prediction with use of continuous glucose and physical activity monitoring data: The Maastricht Study
Source: PLoS One. 2021 Jun 24;16(6):e0253125. doi: 10.1371/journal.pone.0253125 (PMC8224858; doi:10.1371/journal.pone.0253125)
Supplement: S4 Fig — (DOCX) [file pone.0253125.s004.docx]

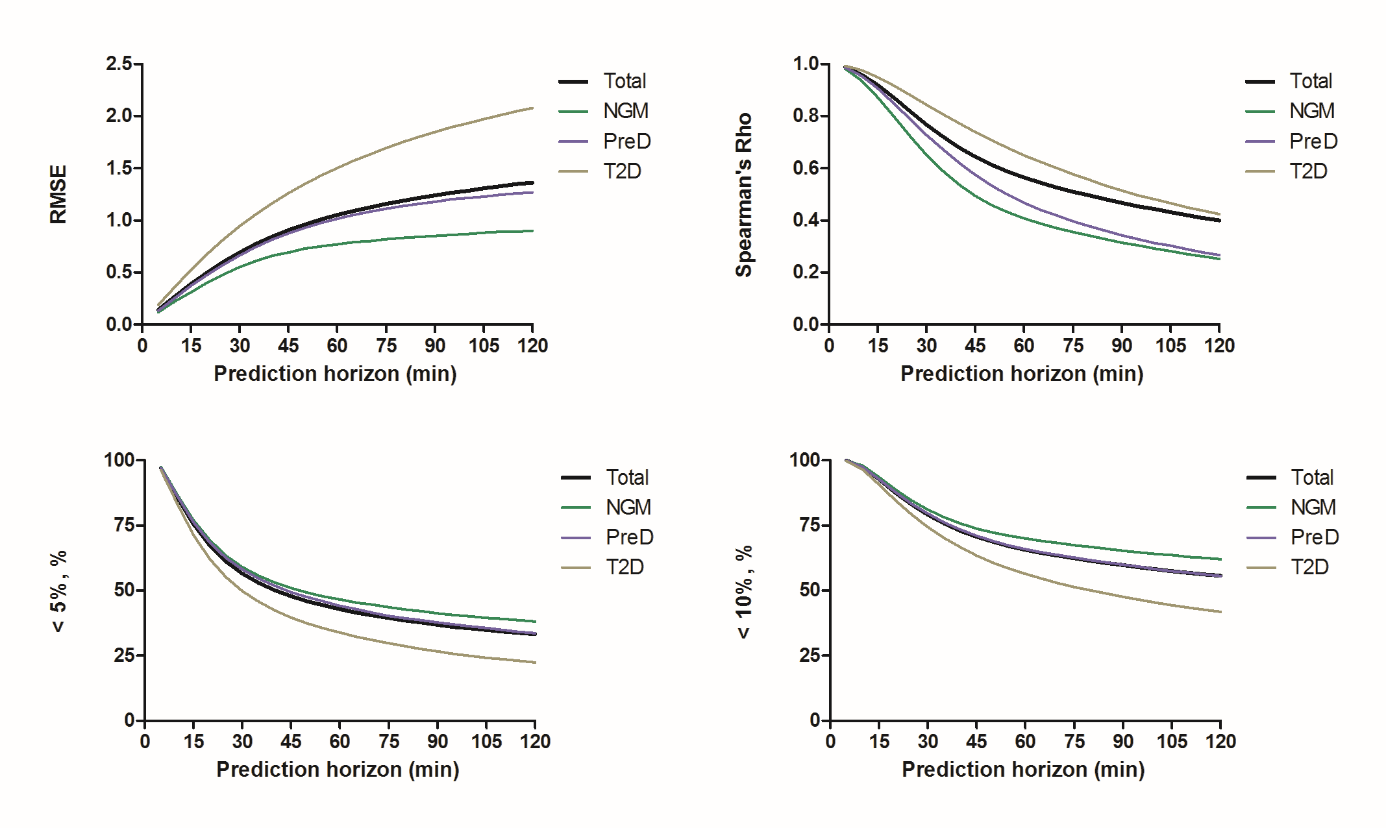


**S4 Fig. Performance characteristics of a prediction model using t_0_ as predictor across time horizons between 0 and 120 minutes**

An extended analysis of model performance using t_0_ glucose value as predictor was carried out for normal glucose metabolism (NGM), prediabetes (PreD) and individuals with type 2 diabetes (T2D). Models were evaluated using RMSE (root-mean-square error; upper-left), Spearman’s rank correlation coefficient (upper-right) and percentage of predicted values within 5% and 10% of actual glucose values, respectively (lower-left and right).
