# Supplementary material for: Machine learning-based glucose prediction with use of continuous glucose and physical activity monitoring data: The Maastricht Study
Source: PLoS One. 2021 Jun 24;16(6):e0253125. doi: 10.1371/journal.pone.0253125 (PMC8224858; doi:10.1371/journal.pone.0253125)
Supplement: S5 Fig — (DOCX) [file pone.0253125.s005.docx]

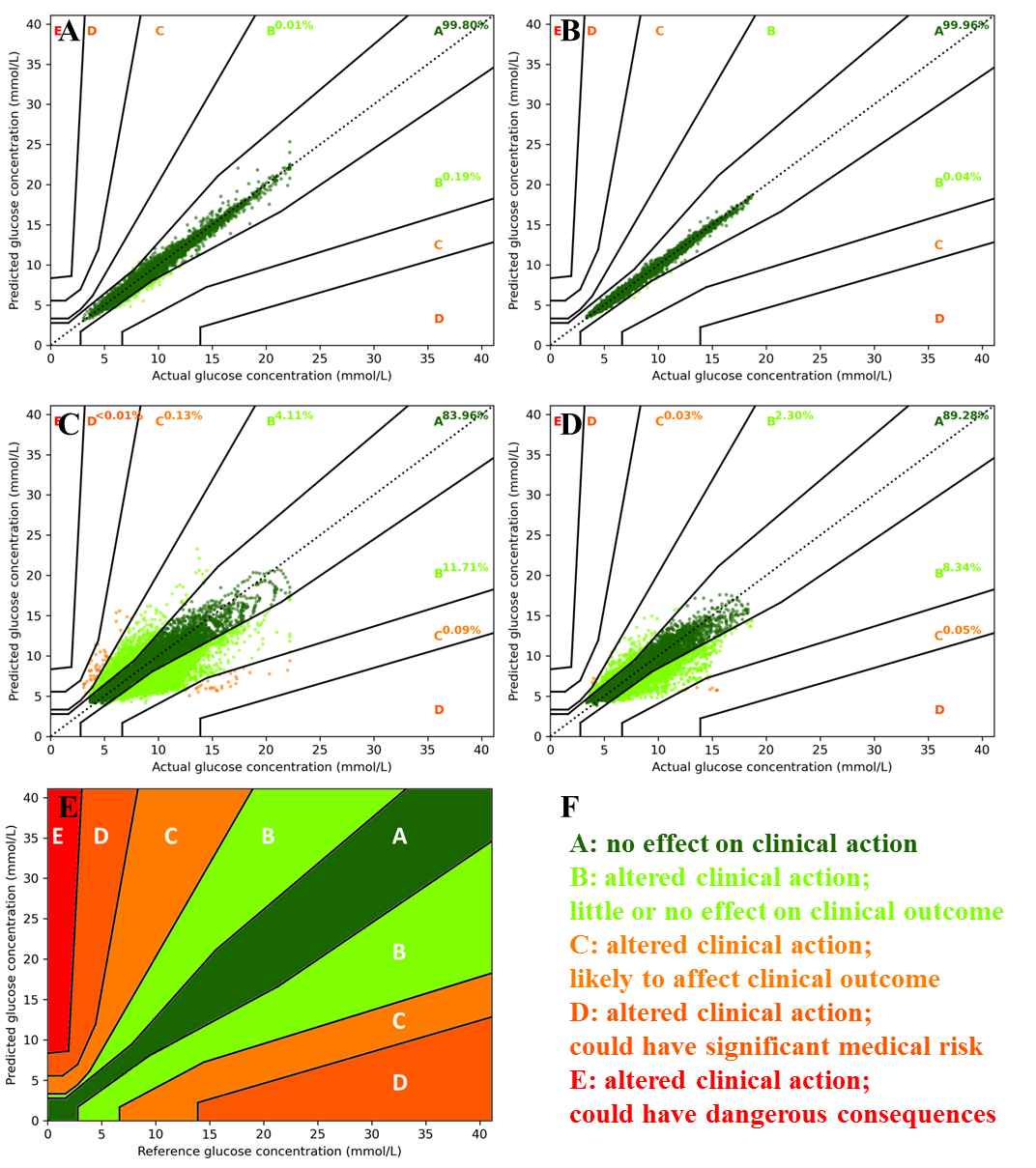


**S5 Fig. Parkes error grid evaluation of glucose prediction safety at time intervals of 15 and 60 minutes**

Assessment of CGM-based glucose prediction safety in individuals with type 2 diabetes (n=43) at 15 minutes (panel A) and 60 minutes (panel C). Assessment of CGM- and accelerometry-based glucose prediction safety in individuals with type 2 diabetes (n=13) at 15 minutes (panel B) and 60 minutes (panel D). Each error zone (panel E) has a different clinical interpretation and consequence (panel F). A horizontal shift towards zone E represents overestimation by the algorithm (higher predicted than actual glucose values); a vertical shift towards zone D represents underestimation (lower predicted than actual glucose values).
