# Supplementary material for: Machine learning-based glucose prediction with use of continuous glucose and physical activity monitoring data: The Maastricht Study
Source: PLoS One. 2021 Jun 24;16(6):e0253125. doi: 10.1371/journal.pone.0253125 (PMC8224858; doi:10.1371/journal.pone.0253125)
Supplement: S1 Table — (DOCX) [file pone.0253125.s006.docx]

**S1 Table. Hyperparameter combinations evaluated in current experiments**

| **Hyperparameter** | **Values considered** |
| --- | --- |
| *Data preprocessing* | |
| Normalization to [0, 1] | On, off |
| Back-propagation window | 15, 30, 60, 120 minutes |
| *Neural Network architecture* | |
| RNN cell type | LSTM, RNN, GRU |
| RNN cell type, bi-directional structure | On, off |
| RNN number of hidden layers | 1, 2, 3 |
| RNN cell size | 128, 64, 32, 16, 8, 4 |
| RNN, activation function | ReLU, leaky ReLU, tanh, sigmoid, ELU |
| Dropout, presence | On, off |
| Dropout | 0.05, 0.1, 0.2, 0.25 |
| *Model training* | |
| Learning rate | 1^e^-2, 1^e^-3, 1^e^-4, 1^e^-5 |
| Learning rate scheduling | On, off |
| Learning rate scheduling decay | 0.5, 0.6, 0.7, 0.8, 0.9, 0.95 |
| Batch size | 64, 128, 256, 512, 1024, 2048, 4096 |
| Optimizer scheme | Adam, NAdam, RMSprop, SGD |
