# Supplementary material for: Machine learning-based glucose prediction with use of continuous glucose and physical activity monitoring data: The Maastricht Study
Source: PLoS One. 2021 Jun 24;16(6):e0253125. doi: 10.1371/journal.pone.0253125 (PMC8224858; doi:10.1371/journal.pone.0253125)
Supplement: S2 Table — (DOCX) [file pone.0253125.s007.docx]

**S2 Table. Final set of hyperparameters for each of the machine learning models**

| **Hyperparameter** | **CGM-based glucose prediction** | **Combined glucose prediction** |
| --- | --- | --- |
| *Data preprocessing* | |  |
| Normalization to [0, 1] | On | On |
| Back-propagation window | 30 minutes | 30 minutes |
| *Neural Network architecture* | |  |
| RNN cell type | LSTM, LSTM, Dense | LSTM, LSTM, Dense |
| RNN cell type, bi-directional structure | Off, off, off | On, off, off |
| RNN number of hidden layers | 3 | 3 |
| RNN cell size | 32, 16, 8 | 64, 32, 8 |
| RNN, activation function | ReLU, ReLU, ReLU | ReLU, ReLU, ReLU |
| Dropout, presence | Off, off, on | Off, off, on |
| Dropout | 0.1 | 0.1 |
| *Model training* | |  |
| Learning rate | 0.001 | 0.001 |
| Learning rate scheduling | On | On |
| Learning rate scheduling decay | 0.005 every 1,000 steps | 0.005 every 1,000 steps |
| Batch size | 1024 | 1024 |
| Optimizer scheme | Adam | Adam |
