# Supplementary material for: Machine learning-based glucose prediction with use of continuous glucose and physical activity monitoring data: The Maastricht Study
Source: PLoS One. 2021 Jun 24;16(6):e0253125. doi: 10.1371/journal.pone.0253125 (PMC8224858; doi:10.1371/journal.pone.0253125)
Supplement: S3 Table — (DOCX) [file pone.0253125.s008.docx]

**S3 Table. Extended baseline characteristics.** Baseline characteristics for overall study population and subgroups normal glucose metabolism NGM) and prediabetes (PreD).

|  | **CGM-based glucose prediction** | | | | **Combined glucose prediction** | | | |
| --- | --- | --- | --- | --- | --- | --- | --- | --- |
| **Characteristic** | **Total (n=851)** | **NGM (n=470)** | **PreD (n=184)** | | **Total (n=540)** | **NGM (n=373)** | **PreD (n=99)** |  |
| Age, years | 59.9 ± 8.7 | 58.2 ± 8.8 | 61.5 ± 8.1 | 59.1 ± 8.7 | | 58.1 ± 8.9 | 60.7 ± 8.5 |  |
| Women, n (%) | 418 (49.1) | 266 (56.6) | 83 (45.1) | 276 (51.1) | | 207 (55.5) | 47 (47.5) |  |
| BMI, kg/m^2^ | 27.2 ± 4.4 | 25.6 ± 3.6 | 28.5 ± 4.4 | 26.5 ± 4.0 | | 25.6 ± 3.5 | 28.8 ± 4.4 |  |
| Newly diagnosed T2D, n (%) | 70 (8.2) | - | - | 35 (6.5) | | - | - |  |
| Fasting plasma glucose, mmol/L | 5.4 [5.0 – 6.2] | 5.1 [4.8 – 5.4] | 6.0 [5.4 – 6.3] | 5.3 [4.9 – 5.8] | | 5.1 [4.8 – 5.4] | 5.9 [5.3 – 6.2] |  |
| 2-h post-load glucose, mmol/L | 6.7 [5.2 – 9.1]* | 5.5 [4.7 – 6.4] | 8.4 [7.5 – 9.2] | 6.2 [5.0 – 7.7] | | 5.5 [4.7 – 6.4] | 8.4 [7.8 – 9.3] |  |
| HbA_1c_, % | 5.7 ± 0.8 | 5.4 ± 0.3 | 5.6 ± 0.4 | 5.6 ± 0.6 | | 5.4 ± 0.3 | 5.5 ± 0.4 |  |
| HbA_1c_, mmol/mol | 39.1 ± 8.3 | 35.4 ± 3.4 | 37.8 ± 4.2 | 37.3 ± 6.2 | | 35.5 ± 3.4 | 37.1 ± 4.4 |  |
| Sensor glucose  Mean, mmol/L | 6.1 [5.7 – 6.7] | 5.8 [5.5 – 6.1] | 6.2 [5.8 – 6.6] | 5.9 [5.6 – 6.4] | | 5.9 [5.5 – 6.1] | 6.2 [5.7 – 6.6] |  |
| SD, mmol/L | 0.84 [0.68 – 1.18] | 0.73 [0.62 – 0.87] | 0.90 [0.74 – 1.13] | 0.79 [0.66 – 1.01] | | 0.73 [0.63 – 0.89] | 0.89 [0.73 – 1.11] |  |
| SD > 1.37 mmol/L, n (%) | 142 (16.7) | 11 (2.3) | 16 (8.7) | 50 (9.3) | | 9 (2.4) | 5 (5.1) |  |
| CV, % | 14.0 [11.6 – 17.6] | 12.6 [10.8 – 14.9] | 14.9 [12.2 – 17.5] | 13.3 [11.2 – 16.8] | | 12.8 [10.9 – 15.2] | 14.7 [12.1 – 17.5] |  |
|  |  |  |  |  | |  |  |  |
| Diabetes medication use, n (%) | 109 (12.8)† | - | - | 27 (4.8) | | - | - |  |
| Insulin | 19 (2.2) | - | - | 4 (0.7) | | - | - |  |
| Metformin | 104 (12.2) | - | - | 27 (5.0) | | - | - |  |
| Sulfonylureas | 21 (2.5) | - | - | 6 (1.1) | | - | - |  |
| Thiazolidinediones | 0 (0) | - | - | 0 (0) | | - | - |  |
| GLP-1 analogs | 3 (0.4) | - | - | 1 (0.2) | | - | - |  |
| DDP-4 inhibitors | 1 (0.1) | - | - | 0 (0) | | - | - |  |
| SGLT-2 inhibitors | 1 (0.1) | - | - | 0 (0) | | - | - |  |
|  |  |  |  |  | |  |  |  |
| Office SBP, mmHg | 133.3 ± 18.0 | 129.3 ± 17.5 | 137.2 ± 19.2 | 132.2 ± 17.9 | | 129.6 ± 17.6 | 138.1 ± 18.6 |  |
| Office DBP, mmHg | 75.2 ± 10.2 | 73.5 ± 9.8 | 76.7 ± 10.3 | 74.7 ± 10.1 | | 73.5 ± 9.8 | 77.2 ± 10.8 |  |
| Antihypertensive medication use, n (%) | 305 (35.9)† | 105 (22.3) | 74 (40.2) | 162 (30.0) | | 84 (22.5) | 37 (37.4) |  |
| Total-to-HDL cholesterol ratio | 3.5 [2.8 – 4.3] | 3.3 [2.8 – 4.4] | 3.8 [3.1 – 4.7] | 3.4 [2.8 – 4.3] | | 3.3 [2.8 – 4.3] | 3.6 [2.9 – 4.5] |  |
| Triglycerides, mmol/L | 1.3 [0.9 – 1.8] | 1.1 [0.8 – 1.5] | 1.4 [1.0 – 2.0] | 1.2 [0.9 – 1.7] | | 1.1 [0.8 – 1.5] | 1.4 [1.0 – 1.9] |  |
| Lipid-modifying medication use, n (%) | 212 (24.9)† | 52 (11.1) | 45 (24.5) | 100 (18.5) | | 38 (10.2) | 23 (23.2) |  |
| Smoking status |  |  |  |  | |  |  |  |
| Never/former/current, n | 327/415/106‡ | 198/210/60 | 62/101/20 | 214/253/70 | | 160/164/47 | 35/52/10 |  |
| Never/former/current, % | 38.6/48.9/12.5 | 42.3/44.9/12.8 | 33.9/55.2/10.9 | 39.9/47.1/13.0 | | 43.1/44.2/12.7 | 35.7/54.1/10.2 |  |

Data are reported as mean ± SD, median [interquartile range], or number (percentage [%]) as appropriate. CGM, continuous glucose monitoring; NGM, normal glucose metabolism; PreD, prediabetes; BMI, body mass index; HbA_1c_, glycated hemoglobin A_1c_; S D, standard deviation; CV, coefficient of variation; GLP-1, glucagon-like peptide-1; DPP-4, dipeptidase-4; SGLT-2, sodium-glucose cotransporter 2; SBP, systolic blood pressure; DBP, diastolic blood pressure; HDL, high-density lipoprotein. * Missing in 38 participants; † missing in one participant; ‡ missing in three participants.
