# Supplementary material for: Machine learning-based glucose prediction with use of continuous glucose and physical activity monitoring data: The Maastricht Study
Source: PLoS One. 2021 Jun 24;16(6):e0253125. doi: 10.1371/journal.pone.0253125 (PMC8224858; doi:10.1371/journal.pone.0253125)
Supplement: S5 Table — (DOCX) [file pone.0253125.s010.docx]

**S5 Table. Extended analysis on time lag between predicted and actual glucose values**

| **Time lag** | | **Total (n=170)** | **NGM (n=92)** | **PreD (n=35)** | **T2D (n=43)** |
| --- | --- | --- | --- | --- | --- |
| **15 minutes** | CGM-based | 0.12 ± 0.18 | 0.08 ± 0.12 | 0.07 ± 0.11 | 0.41 ± 0.92 |
|  | Combined | 0.17 ± 0.11 | 0.11 ± 0.18 | 0.10 ± 0.18 | 0.44 ± 0.38 |
| **60 minutes** | CGM-based | 12.28 ± 6.84 | 7.02 ± 3.46 | 9.03 ± 3.77 | 14.92 ± 11.18 |
|  | Combined | 11.95 ± 7.32 | 7.19 ± 2.87 | 8.75 ± 3.91 | 14.28 ± 9.96 |

*Data are reported as mean ± SD. NGM, normal glucose metabolism; PreD, prediabetes; T2D, type 2 diabetes*

| **Time lag** | | **Total (n=170)** | **NGM (n=92)** | **PreD (n=35)** | **T2D (n=43)** |
| --- | --- | --- | --- | --- | --- |
| **15 minutes** | CGM-based | 0 [0 – 5] | 0 [0 – 0] | 0 [0 – 0] | 0 [0 – 0] |
|  | Combined | 0.50 [0.25 – 0.75] | 0.25 [0.0 – 0.75] | 0.25 [0 – 0.50] | 0.50 [0.25 – 0.75] |
| **60 minutes** | CGM-based | 10 [5 – 15] | 10 [0 – 15] | 10 [5 – 15] | 15 [5 – 20] |
|  | Combined | 9.50 [4.25 – 16.50] | 6.75 [4.25 – 9.50] | 7.50 [4.50 – 10.75] | 14.50 [6.75 – 21.50] |

*Data are reported as median [IQR]. NGM, normal glucose metabolism; PreD, prediabetes; T2D, type 2 diabetes*
