# Supplementary material for: Machine learning-based glucose prediction with use of continuous glucose and physical activity monitoring data: The Maastricht Study
Source: PLoS One. 2021 Jun 24;16(6):e0253125. doi: 10.1371/journal.pone.0253125 (PMC8224858; doi:10.1371/journal.pone.0253125)
Supplement: S6 Table — (DOCX) [file pone.0253125.s011.docx]

**S6 Table. Extended analysis of model performance with t_0_ glucose value as predictor**

| **Prediction with t_0_** | | **Total (n=170)** | **NGM (n=92)** | **PreD (n=35)** | **T2D (n=43)** |
| --- | --- | --- | --- | --- | --- |
| **15 minutes** | RMSE, mmol/L | 0.158 [0.154 – 0.161] | 0.238 [0.233 – 0.343] | 0.151 [0.149 – 0.153] | 0.232 [0.227 – 0.237] |
|  | < 5% , % | 93.56 [93.50 - 93.62] | 92.58 [92.51 – 92.66] | 93.76 [93.71 – 93.80] | 92.65 [92.57 – 97.73] |
|  | < 10% , % | 99.47 [99.42 – 99.52] | 99.01 [98.98 – 99.05] | 99.65 [99.62 – 99.68] | 99.08 [99.04 – 99.12] |
|  | Rho | 0.951 [0.948 – 0.954] | 0.973 [0.967 – 0.980] | 0.953 [0.950 – 0.957] | 0.974 [0.968 – 0.979] |
| **60 minutes** | RMSE, mmol/L | 0.501 [0.498 – 0.505] | 0.602 [0.594 – 0.610] | 0.503 [0.495 – 0.510] | 0.599 [0.594 – 0.604] |
|  | < 5% , % | 74.19 [74.11 – 74.26] | 69.48 [69.39 – 69.56] | 75.02 [74.95 – 75.08] | 70.01 [69.95 – 70.07] |
|  | < 10% , % | 89.89 [89.82 – 89.97] | 87.43 [87.36 – 87.50] | 89.25 [89.19 – 89.31] | 88.20 [88.09 – 88.30] |
|  | Rho | 0.699 [0.697 – 0.702] | 0.732 [0.727 – 0.738] | 0.701 [0.697 – 0.705] | 0.739 [0.732 – 0.747] |

*Data are reported as mean [95% confidence interval]. NGM, normal glucose metabolism; PreD, prediabetes; T2D, type 2 diabetes; RMSE, root-mean-square error; < 5%, percentage of predicted values within 5% of actual glucose values; < 10%, percentage of predicted values within 10% of actual glucose values; rho, Spearman’s rank correlation coefficient.*
