# Supplementary material for: Machine learning-based glucose prediction with use of continuous glucose and physical activity monitoring data: The Maastricht Study
Source: PLoS One. 2021 Jun 24;16(6):e0253125. doi: 10.1371/journal.pone.0253125 (PMC8224858; doi:10.1371/journal.pone.0253125)
Supplement: S7 Table — (DOCX) [file pone.0253125.s012.docx]

**S7 Table. Model performance stratified by day and night**

| **15 minutes** | | **CGM-based glucose prediction** | | **Combined glucose prediction** | |
| --- | --- | --- | --- | --- | --- |
|  |  | Total (n=170) | T2D (n=43) | Total (n=109) | T2D (n=13) |
| **Day** | RMSE, mmol/L | 0.199 [0.196 – 0.202] | 0.300 [0.295 – 0.305] | 0.197 [0.193 – 0.201] | 0.287 [0.283 – 0.291] |
|  | < 5% , % | 92.92 [92.85 – 92.99] | 91.97 [91.87 – 92.07] | 92.94 [92.90 – 92.98] | 91.95 [91.92 – 91.98] |
|  | < 10% , % | 99.11 [99.07 – 99.15] | 98.84 [98.72 – 98.95] | 99.17 [99.13 – 99.21] | 98.82 [98.78 – 98.86] |
|  | Rho | 0.955 [0.952 – 0.958] | 0.984 [0.981 – 0.987] | 0.964 [0.962 – 0.966] | 0.986 [0.984 – 0.988] |
| **Night** | RMSE, mmol/L | 0.182 [0.175 – 0.189] | 0.278 [0.273 – 0.283] | 0.178 [0.173 – 0.183] | 0.257 [0.252 – 0.262] |
|  | < 5% , % | 93.08 [93.01 – 93.15] | 92.07 [91.99 – 92.15] | 93.11 [93.07 – 93.15] | 92.14 [92.10 – 92.18] |
|  | < 10% , % | 99.28 [99.20 – 99.36] | 98.94 [98.88 – 99.00] | 99.34 [99.30 – 99.38] | 99.03 [98.98 – 99.08] |
|  | Rho | 0.967 [0.964 – 0.970] | 0.989 [0.986 – 0.992] | 0.974 [0.970 – 0.978] | 0.994 [0.992 – 0.996] |

| **60 minutes** | | **CGM-based glucose prediction** | | **Combined glucose prediction** | |
| --- | --- | --- | --- | --- | --- |
|  |  | Total (n=170) | T2D (n=43) | Total (n=109) | T2D (n=13) |
| **Day** | RMSE, mmol/L | 0.687 [0.683 – 0.691] | 0.775 [0.768 – 0.783] | 0.536 [0.532 – 0.540] | 0.768 [0.760 – 0.776] |
|  | < 5% , % | 68.40 [68.35 – 68.45] | 63.69 [63.57 – 63.81] | 68.87 [68.80 – 68.94] | 64.03 [63.95 – 64.11] |
|  | < 10% , % | 85.27 [85.20 – 85.35] | 83.82 [83.77 – 83.87] | 86.12 [86.07 – 86.17] | 84.08 [84.01 – 84.15] |
|  | Rho | 0.640 [0.629 – 0.651] | 0.703 [0.697 – 0.709] | 0.658 [0.654 – 0.663] | 0.710 [0.705 – 0.715] |
| **Night** | RMSE, mmol/L | 0.497 [0.491 – 0.503] | 0.634 [0.629 – 0.639] | 0.512 [0.503 – 0.521] | 0.633 [0.627 – 0.639] |
|  | < 5% , % | 72.61 [72.56 – 72.66] | 69.42 [69.33 – 69.51] | 71.58 [71.47 – 71.69] | 69.28 [69.21 – 69.35] |
|  | < 10% , % | 89.44 [89.31 – 89.57] | 87.10 [87.01 – 87.20] | 88.78 [88.68 – 88.88] | 87.30 [87.19 – 87.41] |
|  | Rho | 0.793 [0.784 – 0.802] | 0.854 [0.848 – 0.860] | 0.783 [0.780 – 0.786] | 0.861 [0.855 – 0.867] |

*Data are reported as mean [95% confidence interval]. CGM, continuous glucose monitoring; T2D, type 2 diabetes; RMSE, root-mean-square error; < 5%, percentage of predicted values within 5% of actual glucose values; < 10%, percentage of predicted values within 10% of actual glucose values; rho, Spearman’s rank correlation coefficient.*
