# Supplementary material for: Machine learning-based glucose prediction with use of continuous glucose and physical activity monitoring data: The Maastricht Study
Source: PLoS One. 2021 Jun 24;16(6):e0253125. doi: 10.1371/journal.pone.0253125 (PMC8224858; doi:10.1371/journal.pone.0253125)
Supplement: S8 Table — (DOCX) [file pone.0253125.s013.docx]

**S8 Table. Model performance stratified by low versus high glucose variability**

|  | | **CGM-based glucose prediction** | | **Combined glucose prediction** | |
| --- | --- | --- | --- | --- | --- |
|  |  | SD ≤ 1.37 mmol/L  (n=142) | SD > 1.37 mmol/L  (n=28) | SD ≤ 1.37 mmol/L (n=101) | SD > 1.37 mmol/L  (n=8) |
| **15 minutes** | RMSE, mmol/L | 0.179 [0.176 – 0.181] | 0.301 [0.289 – 0.313] | 0.180 [0.177 – 0.183] | 0.288 [0.276 – 0.299] |
|  | < 5% , % | 93.02 [92.99 – 93.05] | 91.88 [91.77 – 92.05] | 93.21 [93.18 – 93.24] | 92.00 [91.92 – 92.08] |
|  | < 10% , % | 99.25 [99.22 – 99.28] | 98.79 [98.76 – 98.84] | 99.30 [99.29 – 99.32] | 98.82 [98.73 – 98.91] |
|  | Rho | 0.960 [0.959 – 0.962] | 0.983 [0.980 – 0.986] | 0.965 [0.964 – 0.967] | 0.992 [0.988 – 0.996] |
| **60 minutes** | RMSE, mmol/L | 0.549 [0.542 – 0.555] | 0.711 [0.699 – 0.724] | 0.559 [0.552 – 0.565] | 0.710 [0.700 – 0.719] |
|  | < 5% , % | 71.04 [70.89 – 71.21] | 65.33 [65.19 – 66.46] | 71.81 [71.77 – 71.85] | 66.17 [66.09 – 66.23] |
|  | < 10% , % | 89.19 [89.15 – 89.22] | 84.42 [84.28 – 84.56] | 90.01 [89.95 – 90.06] | 85.25 [85.11 – 85.39] |
|  | Rho | 0.701 [0.700 – 0.702] | 0.741 [0.737 – 0.745] | 0.723 [0.719 – 0.728] | 0.801 [0.784 – 0.817] |

*Data are reported as mean [95% confidence interval]. CGM, continuous glucose monitoring; SD, standard deviation; RMSE, root-mean-square error; < 5%, percentage of predicted values within 5% of actual glucose values; < 10%, percentage of predicted values within 10% of actual glucose values; rho, Spearman’s rank correlation coefficient.*
