# Supplementary material for: Machine learning-based glucose prediction with use of continuous glucose and physical activity monitoring data: The Maastricht Study
Source: PLoS One. 2021 Jun 24;16(6):e0253125. doi: 10.1371/journal.pone.0253125 (PMC8224858; doi:10.1371/journal.pone.0253125)
Supplement: S9 Table — (DOCX) [file pone.0253125.s014.docx]

**S9 Table. Extended analysis of model performance in the OhioT1DM Dataset**

|  | | **15 minutes** | **30 minutes** | **60 minutes** |
| --- | --- | --- | --- | --- |
| **Main model** | RMSE, mmol/L | 0.689 [0.685 – 0.693] | 1.189 [1.183 – 1.195] | 1.918 [1.910 – 1.926] |
|  | < 5% , % | 61.84 [61.64 – 61.04] | 39.10 [38.86 – 39.34] | 22.28 [22.01 – 22.65] |
|  | < 10% , % | 86.02 [85.83 – 86.21] | 64.72 [64.45 – 64.99] | 40.19 [39.89 – 40.50] |
|  | Rho | 0.908 [0.905 – 0.911] | 0.792 [0.789 – 0.795] | 0.605 [0.602 – 0.608] |
| **Optimized model** | RMSE, mmol/L | 0.426 [0.422 – 0.430] | 1.046 [1.039 – 1.052] | 1.733 [1.725 – 1.741] |
|  | < 5% , % | 72.22 [71.95 – 72.47] | 48.99 [48.77 – 49.22] | 39.85 [39.55 – 40.15] |
|  | < 10% , % | 91.22 [91.01 – 91.43] | 71.48 [71.24 – 71.73] | 49.81 [49.48 – 50.14] |
|  | Rho | 0.948 [0.946 – 0.950] | 0.886 [0.884 – 0.888] | 0.689 [0.686 – 0.692] |

*Data are reported as mean [95% confidence interval]. Main model: CGM-based model trained on main study population; Optimized model; main CGM-based model trained on main study population and portion of data from individuals with type 1 diabetes; RMSE, root-mean-square error; < 5%, percentage of predicted values within 5% of actual glucose values; < 10%, percentage of predicted values within 10% of actual glucose values; rho, Spearman’s rank correlation coefficient.*
