# Supplementary material for: Machine learning-based glucose prediction with use of continuous glucose and physical activity monitoring data: The Maastricht Study
Source: PLoS One. 2021 Jun 24;16(6):e0253125. doi: 10.1371/journal.pone.0253125 (PMC8224858; doi:10.1371/journal.pone.0253125)
Supplement: S1 File — (DOCX) [file pone.0253125.s015.docx]

**S1 File. Background information on machine learning models reviewed in current study**

We conducted a comparison of available algorithms on the continuous glucose prediction task. We considered the following algorithms:

- **ARIMA:** an autoregressive integrated moving average (ARIMA) model is an adaptation of the autoregressive moving average (ARMA) model. These models aim to estimate the time series using two polynomials, one for autoregression (AR), and the other for the moving average (MA). ARIMA models for glucose prediction have widely been described in the literature[1-4]. For the current comparison, we used a ARIMA (p=3, d=0, q=0) model based on previous findings by Otoom et al[1].
- **Support vector regression:** support vector regression (SVR) is a generalization of support vector machines (SVM) that work for regression problems. SVR models for the prediction of glucose values have been described in the literature[5, 6]. For the current comparison, we build the SVR model with a Gaussian RFB kernel that was optimized by the differential evolution algorithm as described by Georga et al[5].
- **Gradient-boosting trees:** gradient-boosting trees have shown superior performances in the medical domain, but lack the capability to aggregate information over time. For baseline comparison, we used the LightGBM[7] implementation with a learning rate of 0.01, a maximum number of trees of 500, and a maximum depth of each base learner to be 50.
- **Feed-forward neural networks:** feed-forward neural networks do not have the capacity to aggregate the information about past glucose status of an individual over time. Yet, they are relatively simple networks which have previously been employed for glucose[8-10]. We considered shallow and deep multi-layer perceptron (MLP) neural networks. The shallow MLP consisted of one hidden layer (ReLU) with 16 neurons. The deep MLP consisted of three hidden layers (ReLU) with 64, 32, and 16 neurons, respectively. Both networks were trained using the Adam optimizer scheme with a learning rate of 0.001.
- **Recurrent neural networks:** recurrent neural networks are a type of neural networks with the capacity to explicitly model information over time. These type of neural networks are designed to work with temporal data such as glucose and physical activity data. Although we acknowledge the broad availability of neural network architectures[11-15], we only considered recurrent neural networks (RNN), and long-short term memory (LSTM) networks for the baseline comparison. Both models consisted of one RNN layer (either RNN or LSTM) with 32 neurons, followed by a Dense layer of 8 neurons. Both networks were trained using the Adam optimizer scheme with a learning rate of 0.001.

**References**

1. Otoom M, Alshraideh H, Almasaeid HM, Lopez-de-Ipina D, Bravo J. Real-Time Statistical Modeling of Blood Sugar. J Med Syst. 2015;39(10):123. Epub 2015/08/26. doi: 10.1007/s10916-015-0301-8. PubMed PMID: 26303151.

2. Yang J, Li L, Shi Y, Xie X. An ARIMA Model With Adaptive Orders for Predicting Blood Glucose Concentrations and Hypoglycemia. IEEE J Biomed Health Inform. 2019;23(3):1251-60. Epub 2018/07/12. doi: 10.1109/JBHI.2018.2840690. PubMed PMID: 29993728.

3. Sun Q, Jankovic MV, Bally L, Mougiakakou SG. Predicting Blood Glucose with an LSTM and Bi-LSTM Based Deep Neural Network. arXiv e-prints [Internet]. 2018 September 01, 2018. Available from: <https://ui.adsabs.harvard.edu/abs/2018arXiv180903817S>.

4. Rodriguez-Rodriguez I, Chatzigiannakis I, Rodriguez JV, Maranghi M, Gentili M, Zamora-Izquierdo MA. Utility of Big Data in Predicting Short-Term Blood Glucose Levels in Type 1 Diabetes Mellitus Through Machine Learning Techniques. Sensors (Basel). 2019;19(20). Epub 2019/10/19. doi: 10.3390/s19204482. PubMed PMID: 31623111; PubMed Central PMCID: PMCPMC6833040.

5. Georga EI, Protopappas VC, Ardigo D, Polyzos D, Fotiadis DI. A glucose model based on support vector regression for the prediction of hypoglycemic events under free-living conditions. Diabetes Technol Ther. 2013;15(8):634-43. Epub 2013/07/16. doi: 10.1089/dia.2012.0285. PubMed PMID: 23848178.

6. Hamdi T, Ben Ali J, Di Costanzo V, Fnaiech F, Moreau E, Ginoux J-M. Accurate prediction of continuous blood glucose based on support vector regression and differential evolution algorithm. Biocybernetics and Biomedical Engineering. 2018;38(2):362-72. doi: <https://doi.org/10.1016/j.bbe.2018.02.005>.

7. Ke G, Meng Q, Finley T, Wang T, Chen W, Ma W, et al. LightGBM: A Highly Efficient Gradient Boosting Decision Tree. 2017:3146--54.

8. Allam F, Nossair Z, Gomma H, Ibrahim I, Salam MA-e, editors. Prediction of subcutaneous glucose concentration for type-1 diabetic patients using a feed forward neural network. The 2011 International Conference on Computer Engineering & Systems; 2011 29 Nov.-1 Dec. 2011.

9. Zarkogianni K, Mitsis K, Litsa E, Arredondo MT, Ficomicron G, Fioravanti A, et al. Comparative assessment of glucose prediction models for patients with type 1 diabetes mellitus applying sensors for glucose and physical activity monitoring. Med Biol Eng Comput. 2015;53(12):1333-43. Epub 2015/06/08. doi: 10.1007/s11517-015-1320-9. PubMed PMID: 26049412.

10. Perez-Gandia C, Facchinetti A, Sparacino G, Cobelli C, Gomez EJ, Rigla M, et al. Artificial neural network algorithm for online glucose prediction from continuous glucose monitoring. Diabetes Technol Ther. 2010;12(1):81-8. Epub 2010/01/20. doi: 10.1089/dia.2009.0076. PubMed PMID: 20082589.

11. Hochreiter S, Schmidhuber J. Long Short-Term Memory. Neural Comput. 1997;9(8):1735-80. doi: 10.1162/neco.1997.9.8.1735.

12. Sherstinsky A. Fundamentals of Recurrent Neural Network (RNN) and Long Short-Term Memory (LSTM) Network. arXiv e-prints. 2018:arXiv:1808.03314.

13. Staudemeyer RC, Rothstein Morris E. Understanding LSTM -- a tutorial into Long Short-Term Memory Recurrent Neural Networks. arXiv e-prints [Internet]. 2019 September 01, 2019. Available from: <https://ui.adsabs.harvard.edu/abs/2019arXiv190909586S>.

14. Chung J, Gulcehre C, Cho K, Bengio Y. Empirical Evaluation of Gated Recurrent Neural Networks on Sequence Modeling. arXiv e-prints. 2014:arXiv:1412.3555.

15. Schuster M, Paliwal K. Bidirectional recurrent neural networks. Signal Processing, IEEE Transactions on. 1997;45:2673-81. doi: 10.1109/78.650093.

16. Li K, Liu C, Zhu T, Herrero P, Georgiou P. GluNet: A Deep Learning Framework for Accurate Glucose Forecasting. IEEE J Biomed Health Inform. 2020;24(2):414-23. Epub 2019/08/02. doi: 10.1109/JBHI.2019.2931842. PubMed PMID: 31369390.
