# Supplementary material for: Machine learning-based glucose prediction with use of continuous glucose and physical activity monitoring data: The Maastricht Study
Source: PLoS One. 2021 Jun 24;16(6):e0253125. doi: 10.1371/journal.pone.0253125 (PMC8224858; doi:10.1371/journal.pone.0253125)
Supplement: S2 File — (DOCX) [file pone.0253125.s016.docx]

**S2 File. Background information on metrics used in the current study**

In the current study we used several metrics to assess the performance of our models. In this paragraph we will describe each of them briefly and also provide their mathematical definition.

- Root-mean-square error (RMSE): the RMSE is an average error term which is in the order of the predicted / measured variable. It also can be interpreted as the standard deviation of the prediction errors. The formula of RMSE is as follows:

$$RMSE= \sqrt{\sum_{i=1}^{n} \frac{\left( \hat{y}_{i}- y_{i} \right)^{2}}{n}}$$

*with* $\hat{y}_{1}, \hat{y}_{2}, \hat{y}_{n}$ *depicting the predicted values;* $y_{1,}y_{2}, y_{n}$ *depicting the real values and* $n$ *representing the total number of predictions*

- Correlation: correlation is a measure of how well the relationship between two variables is. In this study, we deal with non-parametrically distributed data and therefore use Spearman’s rank correlation coefficient (rho) which tries to describe the relationship of two variables using a monotonic function:

$$\rho= \frac{S_{xy}}{S_{x}S_{y}}= \frac{\frac{1}{n}\sum_{i=1}^{n} \left( \left( R\left( x_{i} \right)-\overline{R(x)} \right)\cdot\left( R\left( y_{i} \right)- \overline{R(y)} \right) \right)}{\sqrt{\left( \frac{1}{n}\sum_{i=1}^{n} \left( R\left( x_{i} \right)- \overline{R\left( x \right)} \right)^{2} \right)\cdot\left( \frac{1}{n}\sum_{i=1}^{n} \left( R\left( y_{i} \right)- \overline{R\left( y \right)} \right)^{2} \right)}}$$

*with* $x_{1,}x_{2}, x_{n}$ *depicting the predicted values;* $y_{1,}y_{2}, y_{n}$ *depicting the real values and* $n$ *representing the total number of predictions*

- Time lag: time lag is a measure of the time shift between the actual and predicted glucose profile which results in the highest cross correlation coefficient between them [1, 2]:

$$\tau_{delay}= \arg\max_{k} (\check{y_{k}}\left( k | k-PH \right)*y\left( k \right))$$

with$y$ depicting the real values; $\hat{y}_{k}$ depicting the predicted values and PH depicting the prediction horizon

**References**

1. Perez-Gandia C, Facchinetti A, Sparacino G, Cobelli C, Gomez EJ, Rigla M, et al. Artificial neural network algorithm for online glucose prediction from continuous glucose monitoring. Diabetes Technol Ther. 2010;12(1):81-8. Epub 2010/01/20. doi: 10.1089/dia.2009.0076. PubMed PMID: 20082589.

2. Li K, Liu C, Zhu T, Herrero P, Georgiou P. GluNet: A Deep Learning Framework for Accurate Glucose Forecasting. IEEE J Biomed Health Inform. 2020;24(2):414-23. Epub 2019/08/02. doi: 10.1109/JBHI.2019.2931842. PubMed PMID: 31369390.
