# Supplementary material for: Machine learning-based glucose prediction with use of continuous glucose and physical activity monitoring data: The Maastricht Study
Source: PLoS One. 2021 Jun 24;16(6):e0253125. doi: 10.1371/journal.pone.0253125 (PMC8224858; doi:10.1371/journal.pone.0253125)
Supplement: Attachment — Submitted filename: Response to Reviewers.docx [file pone.0253125.s018.docx]

We would like to thank the reviewers for their feedback on our study and for the time spent on our manuscript. Please find below our point-by-point rebuttal.

**Reviewer #1**

**The paper has been improved significantly. All my comments have been addressed with clear explanation and all updates have been shown explicitly in the paper.**

We once more would like to thank the reviewer for his/her positive feedback on our study and for the time spent on our manuscript.

**Reviewer #3**

**I think that my concerns have not been addressed. The paper has little interest for the reader of the journal. In the case of people not working in the field, the contribution is so poor that no extrapolation to other works can be done. On the other hand for people working on this problem, conclusion are known, statistical validation is not made and conclusions are not fully supported by experiments.**

We respectfully disagree with the comments made by the reviewer. We would like to reiterate the main aims of our study. First, we investigated to what extent glucose values can be accurately predicted at 15- and 60-minute intervals, while using the best performing ML-based model in a large sample of participants with either normal glucose metabolism (NGM), prediabetes, or type 2 diabetes. Such large scale data has not yet been used in the context of a glucose prediction study. Second, we assessed whether prediction could be further improved by incorporation of accelerometer-measured physical activity data. Such a large-scale combination of simultaneously collected continuous glucose monitoring and activity tracker has not yet been used. Finally, we augmented the (clinical) interest of our study by including model validation in a type 1 diabetes dataset in the revised version of the manuscript, for which the reviewer is greatly acknowledged. In conclusion, the combination of our large cohort of individuals with NGM, prediabetes or type 2 diabetes as well as the examination of glucose measurements combined with physical activity data has never been described to date and can, therefore, be of great interest to the readers of PLOS ONE.

**The inclusion of T1D patients is forced in my opinion and does not make much sense with the other results.**

We chose to include individuals with type 1 diabetes in order to examine the performance of our models in this subgroup as part of a proof-of-concept analysis. As described in our Discussion, we agree that further, comprehensive evaluation is necessary in order to examine the real clinical benefit and performance of our models in individuals with type 1 diabetes. Notably, previous studies have also employed this dataset in order to validate their models (1-7).

**My questions are again the same 2. Secondly, no new techniques are proposed,....
Analyses are mere description of the results, see for instance lines 323 to 329:**

***Additional analyses***

**To further obtain insights into our model predictions, we assessed performance metrics stratified by day and night (S8 Table). Fifteen-minute predictions did not materially differ between day and night. By contrast, accuracy of 60-minute predictions was lower during the day than at night. In addition, we stratified the results by high or low glucose variability (i.e., SD cut-off of 1.37 mmol/L) (S9 Table). Model performance was slightly lower at higher glucose variability, at both time intervals of 15 and 60 minutes.**

We again would like to stress that the main objective of the current work is not the technological advancement of algorithms per se. We certainly have the ambition to develop our models further (e.g., by using more complex or innovative algorithms), but view such endeavors to be out of scope for the current manuscript.

**4. Experimental results are not useful as they are presented in the paper. All the techniques are summarized in just one table and no discrimination among them is done.**

We assume the reviewer is referencing to the baseline comparison of various statistical and machine learning models as depicted in S1 Table. In line with our previous comment, we would like to point out that we did not aim to achieve technological advancements. Hence, we intended to find the best machine learning-based prediction model as a means to an end, not as a goal in itself. After we concluded that LSTM performed best, especially at a prediction interval of 60 minutes, there was no need to further address or compare all other possible techniques. As such, the comprehensive baseline evaluation of different statistical and machine learning models can even be considered a strength of our study.

**References**

1.	Marling C, Bunescu RC, editors. The OhioT1DM Dataset For Blood Glucose Level Prediction. KHD@IJCAI; 2018.

2.	Marling C, Bunescu RC, editors. The OhioT1DM Dataset For Blood Glucose Level Prediction: Update 2020. KHD@IJCAI; 2020.

3.	Pappada SM, Owais MH, Cameron BD, Jaume JC, Mavarez-Martinez A, Tripathi RS, et al. An Artificial Neural Network-based Predictive Model to Support Optimization of Inpatient Glycemic Control. Diabetes Technol Ther. 2020;22(5):383-94.

4.	Li K, Liu C, Zhu T, Herrero P, Georgiou P. GluNet: A Deep Learning Framework for Accurate Glucose Forecasting. IEEE J Biomed Health Inform. 2020;24(2):414-23.

5.	Chen J, Li K, Herrero P, Zhu T, Georgiou P, editors. Dilated Recurrent Neural Network for Short-time Prediction of Glucose Concentration. KHD@IJCAI; 2018.

6.	Martinsson J, Schliep A, Eliasson B, Meijner C, Persson S, Mogren O. Automatic blood glucose prediction with confidence using recurrent neural networks. 3rd International Workshop on Knowledge Discovery in Healthcare Data, KDH@IJCAI-ECAI 2018, 13 July 2018; 20182018. p. 64-8.

7.	Kriventsov S, Lindsey A, Hayeri A. The Diabits App for Smartphone-Assisted Predictive Monitoring of Glycemia in Patients With Diabetes: Retrospective Observational Study. JMIR Diabetes. 2020;5(3):e18660.
