# Supplementary material for: Machine learning-based glucose prediction with use of continuous glucose and physical activity monitoring data: The Maastricht Study
Source: PLoS One. 2021 Jun 24;16(6):e0253125. doi: 10.1371/journal.pone.0253125 (PMC8224858; doi:10.1371/journal.pone.0253125)
Supplement: Attachment — Submitted filename: Response to Reviewers.docx [file pone.0253125.s019.docx]

We would like to thank the reviewers for their feedback on our study and for the time spent on our manuscript. Please find below our point-by-point rebuttal. The page and line numbers refer to the manuscript version with track changes.

**Reviewer #1**

**My all comments have been addressed properly. The only issue is that, Plus One requires all data underlying the findings in the manuscript fully available. It is better to address this issue before publish.**

Data of The Maastricht Study are certainly available to researchers who meet the criteria for access to confidential data. For the safety and privacy of the participants, as requested by law and ethical regulations, strict procedures to obtain data are in place. The implication of this is that the data have been deemed unsuitable for public deposition by The Board of The Maastricht Study, as described in detail under *Data availability* (Page 24, Lines 493-498). This should not be a ground to preclude publication of our work in PLOS ONE. Accordingly, multiple manuscripts that used data from The Maastricht Study and, thus, were under the same restrictions regarding data availability have been published in PLOS ONE(1-5).

**Reviewer #3**

**I really appreciate the efforts of the authors for improving the paper and answering my questions. I would like to explain better which is my opinion about the great potential of the work. What I would expect of such amount of data is to obtain guidelines for selecting and designing better ML (or not ML) algorithms, based on the precision needed, the time for response, the data availability and of course the features of the patient. For me, what it would be useful for the journal readers is a combination of tables 1 and 2 with information provided as supporting information. As the supporting information is going to be publish, a summary of this information should be included in the paper, in order to highlight the insights of this study. I would like to see a Table with a summary of the supporting information in the main paper. The paper is is a very interesting work**

As suggested by the reviewer, we have now included the baseline comparison of statistical and machine learning models for glucose prediction in our main manuscript as Table 1 (Page 12, Lines 223-225). Accordingly, we made various changes in the methods section of our main manuscript (Page 10, Lines 195-205; Page 22, Lines 439-441) and supplementary information (Page 4, Lines 92-98).

**References**

1.	de Rooij BH, van der Berg JD, van der Kallen CJ, Schram MT, Savelberg HH, Schaper NC, et al. Physical Activity and Sedentary Behavior in Metabolically Healthy versus Unhealthy Obese and Non-Obese Individuals - The Maastricht Study. PLoS One. 2016;11(5):e0154358.

2.	Sorensen BM, Houben A, Berendschot T, Schouten J, Kroon AA, van der Kallen CJH, et al. Cardiovascular risk factors as determinants of retinal and skin microvascular function: The Maastricht Study. PLoS One. 2017;12(10):e0187324.

3.	Elissen AMJ, Hertroijs DFL, Schaper NC, Bosma H, Dagnelie PC, Henry RM, et al. Differences in biopsychosocial profiles of diabetes patients by level of glycaemic control and health-related quality of life: The Maastricht Study. PLoS One. 2017;12(7):e0182053.

4.	Martens RJH, van der Berg JD, Stehouwer CDA, Henry RMA, Bosma H, Dagnelie PC, et al. Amount and pattern of physical activity and sedentary behavior are associated with kidney function and kidney damage: The Maastricht Study. PLoS One. 2018;13(4):e0195306.

5.	Consolazio D, Koster A, Sarti S, Schram MT, Stehouwer CDA, Timmermans EJ, et al. Neighbourhood property value and type 2 diabetes mellitus in the Maastricht study: A multilevel study. PLoS One. 2020;15(6):e0234324.
